# Health state utility values for metastatic pancreatic cancer using a composite time trade-off based on the vignette-based approach in Japan

**DOI:** 10.1186/s13561-022-00413-8

**Published:** 2022-12-24

**Authors:** Takumoto Yuki, Sasahara Yuriko, Narimatsu Hirohito, Murata Tatsunori, Akazawa Manabu

**Affiliations:** 1 Department of Public Health and Epidemiology, Meiji Pharmaceutical University, 2-522-1 Noshio, Kiyose, Tokyo, Japan; 2 Center for Outcomes Research and Economic Evaluation for Health, National Institute of Public Health, Saitama, Japan; 3 Department of Medical Oncology, Yamagata Prefectural Central Hospital, Yamagata, Japan; 4 Department of Genetic Medicine, Kanagawa Cancer Center, Yokohama, Kanagawa Japan; 5 Cancer Prevention and Cancer Control Division, Kanagawa Cancer Center Research Institute, Yokohama, Kanagawa Japan; 6 Graduate School of Health Innovation, Kanagawa University of Human Services, Yokohama, Kanagawa Japan; 7 CRECON Medical Assessment Inc, Tokyo, Japan

**Keywords:** Utility, Metastatic pancreatic cancer, Health related quality of life, Time trade-off, Vignette-based method

## Abstract

**Backgrounds:**

Limited information is available on the utility values of metastatic pancreatic cancer, focusing on different health statuses, selected chemotherapy, and related grades 1/2 and 3/4 adverse events (AEs). We evaluated Japanese societal-based health-related utility values for metastatic pancreatic cancer by considering different grade toxicities commonly associated with chemotherapy using the vignette-based method.

**Methods:**

We developed health status scenarios for patients with metastatic pancreatic cancer undergoing chemotherapy and conducted utility research using the developed scenarios in four steps: ‘literature review,’ ‘exploratory interview,’ ‘content validation’, and ‘utility research’. In the development process, to consider the impact of AEs of chemotherapy for metastatic pancreatic cancer on health state utility values, we selected neutropenia, febrile neutropenia, diarrhea, nausea and vomiting, and neuropathy as representative AEs. Each AE was classified as either grade 1/2 or 3/4. We confirmed our created scenarios through cognitive interviews with the general population and clinical experts to validate the content. Finally, we developed 11 scenarios for using ‘utility research,’ evaluated in a societal-based valuation study using the face-to-face method. Participants for ‘utility research’ were the general population, and they evaluated these scenarios in the composite time trade-off (cTTO) and visual analog scale (VAS) of the European quality of life (EuroQol) valuation technology to derive health state utility scores.

**Results:**

Of 220 responders who completed this survey, 201 were adapted into the analysis population. Stable disease with no AEs (reference state) had a mean utility value of 0.653 using cTTO. The lowest mean utility score in the stable state was 0.242 (stable disease + grade 3/4 vomiting). VAS results ranged from 0.189 to 0.468, depending on the various grades of AEs in stable disease. In addition, grade 3/4 AEs and grade 1/2 nausea/vomiting were associated with significantly greater disutility. Utility values were also strongly influenced by the direct impact of AE on physical symptoms, severity and their experience. In addition, 95.9% of the respondents agreed that they understood the questions in the post-response questionnaire.

**Conclusions:**

We clarified the health state utility values of patients with metastatic pancreatic cancer based on the general population in Japan. The effect on utilities should be considered not only for serious AEs, but also for minor AEs.

**Supplementary Information:**

The online version contains supplementary material available at 10.1186/s13561-022-00413-8.

## Background

Pancreatic cancer (PC), the leading cause of cancer-related deaths in developed countries, is one of the deadliest cancers worldwide [1, 2]. In Japan, PC has the fourth highest site-specific cancer mortality rate [3–5]. As PC is asymptomatic and difficult to detect early, it is often diagnosed only at an advanced or metastatic stage [6]. The National Comprehensive Cancer Network (NCCN) and 2019 Japan Pancreas Society guidelines recommend systematic chemotherapy administration for treating metastatic PC (MPC) [7, 8].

The therapeutic aim of systematic chemotherapy for MPC is to prolong prognosis and maintain and improve the quality of life (QOL) [7, 9, 10]. Although systematic chemotherapy can prolong survival, it can cause various adverse events (AEs), including peripheral neuropathy, diarrhea and nausea [11–14]. Besides pain caused by cancer itself, PC is often associated with AEs that are sufficiently severe to require treatment discontinuation. Therefore, various medical care and chemotherapy management are required, depending on disease state and AEs severity [15, 16]. The psychological, social, and economic problems of cancer patients are also severe [17]. With the development of supportive medicines to alleviate physical pain, impact on family work, social activities, and financial burden of cancer patients may outweigh physical pain sometimes [18, 19]. Therefore, it is necessary to assess type, extent, and frequency of safety risks as well as impact of disease and safety endpoints on patients’ health-related QOL (HRQoL) when deciding whether to implement or continue systematic chemotherapy.

There are two main HRQoL assessment types. In preference-based measures (PBMs), measurement results can be quantified as utility values. In non-PBMs, health status in a specific disease can be measured in detail [20], and detailed and specific information on HRQoL of the targeted disease can be collected. Examples for non-PBMs include the Medical Outcomes Study (MOS) short form-36 (SF-36) for patients with mild symptoms, and European Organization for Research and Treatment of Cancer Quality of Life (EORTC-QLQ) for cancer patients [21, 22]. PBMs can indirectly measure utility using a specific questionnaire including the EuroQol 5 dimensions 5-level (EQ-5D-5L), short form 6 dimensions (SF-6D), and health utilities index (HUI) [23–25]. Because indirect utility measurement applies to various diseases, quality-adjusted life years (QALYs) can be calculated from the target disease utility and survival time and used as effectiveness for cost-utility analysis in health technology assessment. Thus, various methods have been developed for evaluating HRQoL. Generally, depending on patients with a targeted disease and study purpose, non-PBMs and PBMs are used to evaluate HRQoL. When it is difficult investigating a patient of interest, utility research by direct methods using vignette-based methods (VBM) is possible. In VBM, health status scenario of patients with specific diseases are created, and the general population is asked to imagine being in that scenario [26]. Such a direct method includes the rating scale (RS) and time trade-off (TTO) [27, 28]. VBM can also be conducted when HRQoL changes over time [29–31]. Utility research on the general population has yielded highly valid and consistent utility values based on appropriate research processes with medical specialists and QOL experts.

In Japan, utility research using EQ-5D has been conducted in patients with advanced PC undergoing chemotherapy [32, 33]. However, MPC patients have a poorer disease status, and it is difficult carrying out large-scale patient surveys among them. Moreover, no studies in Japan have independently and quantitatively evaluated HRQoL impact on MPC disease status and MPC chemotherapy AEs. Furthermore, although MPC chemotherapy has a high patient burden due to AEs, no international studies have evaluated grades 1/2 (G1/2) AEs impact on utility. Therefore, we developed health status scenarios for patients with MPC and conducted a utility research in the general population based on VBM using the developed scenarios, to evaluate impact on MPC disease status HRQoL and chemotherapy-related G1/2 and G3/4 AEs.

## Methods

**Fig. 1:**
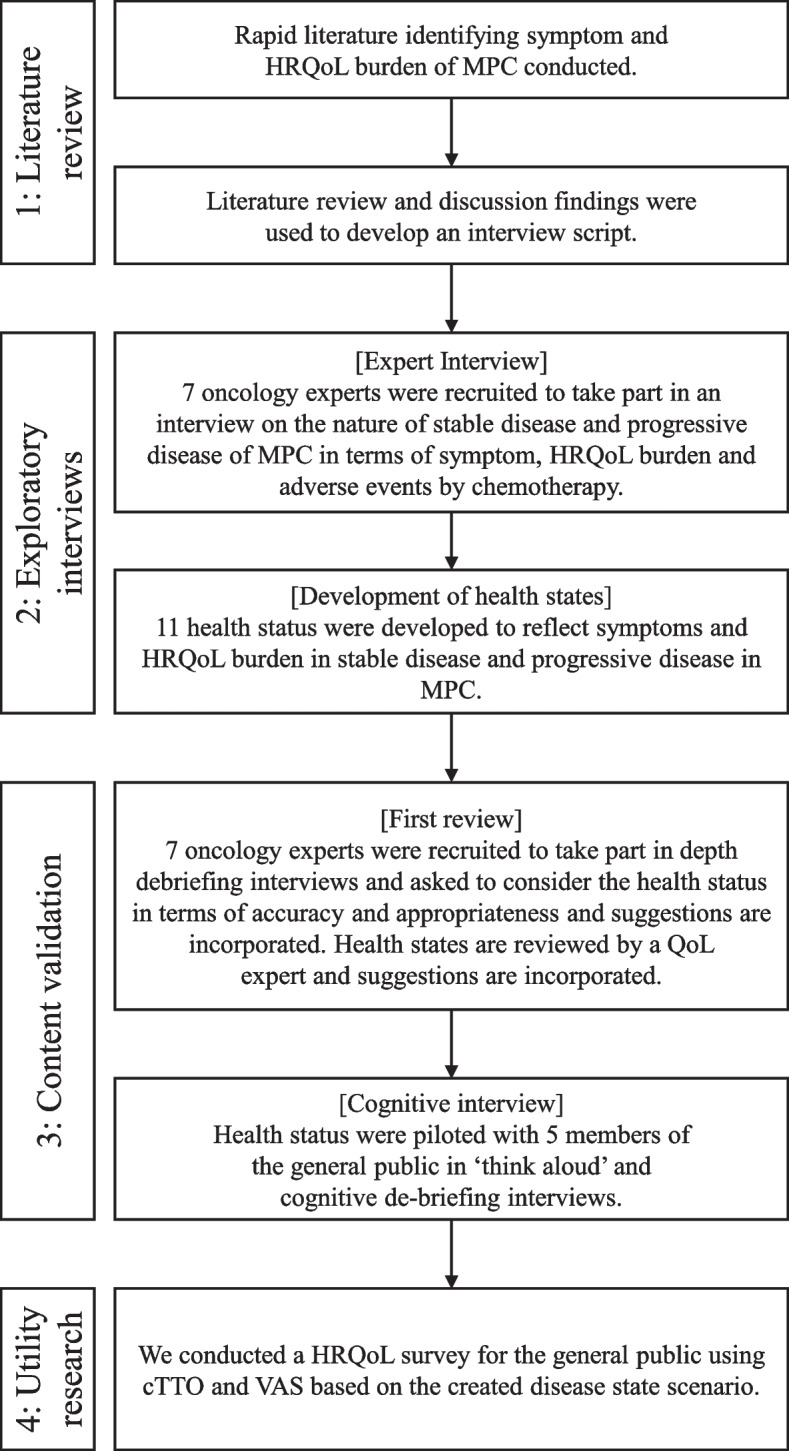
Flow diagram in the development of health statue scenarios for metastatic pancreas cancer. HRQoL, health-related quality of life; MPC, metastatic pancreatic cancer; cTTO, composite time trade-off; VAS, visual analog scale

This study was conducted according to a previous trial on VBM by Louis et al. and ISPOR PRO good research practice task force report [28, 34, 35]. Specifically, the health state scenario development process in PC consisted of four steps: ‘literature review,’ ‘exploratory interviews,’ ‘content validation,’ and ‘utility research’ (Fig. 1). We developed health state scenarios in Steps 1 through 3 and conducted utility research in the general population by composite TTO (cTTO) and visual analog scale (VAS) using health state scenario created in Step 4.

### Step 1: literature review

Literature review was conducted to understand MPC and its impact on HRQoL, and to identify factors affecting HRQoL in PC. Specifically, we collected clinical practice and treatment information of MPC from various guidelines and publications [7, 8]. Regarding MPC, 78–82, 48%, and 66–84% of patients with PC present with abdominal pain, back pain, and weight loss associated with anorexia and other symptoms, suggesting a significant impact of pain and anorexia [36]. AEs were referenced in a representative clinical trial on first-line systemic chemotherapy for MPC [12, 13]. The Common Terminology Criteria for Adverse Events (CTCAE) versions 4 and 5 were reviewed to define AE grades [37, 38]. Furthermore, we also reviewed EORTC QLQ-C30 items and categories to determine disease-specific impact of cancer on HRQoL, because EORTC-QLQ C30 physical, mental, role, and social components might have significant impacts on HRQoL in MPC patients [39].

### Step 2: exploratory interviews

We conducted ‘expert interview’ and ‘development of health states’ through ‘exploratory interviews.’ ‘Expert interview’ was conducted with three oncology physicians and four oncology pharmacists. During interviews, respondents received information on disease status and treatment-related AEs of MPC from literature review; then, they were interviewed about factors affecting HRQoL. To ensure a common understanding of the wording and amount of text in the disease scenarios used in ‘utility research’, we provided respondents with references to previous HRQoL studies of metastatic breast cancer and metastatic non-small cell lung cancer (NSCLC) scenarios [40, 41]. Next, in the ‘development of health states,’ two oncology physicians drafted disease scenarios for our survey based on the expert interview results. Following the ‘development of health states,’ we classified MPC health status into two categories. ‘Stable disease (SD),’ where the disease was stabilized by chemotherapy, and ‘progressive disease (PD),’ where the disease worsened due to increased tumor diameter or other factors. SD is assumed to include systemic chemotherapy; therefore, we categorized the five AEs (neutropenia, febrile neutropenia, nausea, diarrhea, and peripheral neuropathy) with significant impacts on HRQoL into G1/2 and G3/4, resulting in first draft 11 disease scenarios (Table 1). These five adverse event categories were selected based on interviews with oncology physicians and four oncology pharmacists. Our interviews considered the impact on QoL and the frequency of adverse events that occur during chemotherapy for metastatic pancreatic cancer in Japan.

**Table 1:** List of health status scenario

No	Health status		AE Grade
1	Stable disease	No adverse effects	–
2		Neutropenia	1/2
3		Neutropenia	3/4
4		FN	–
5		Diarrhea	1/2
6		Diarrhea	3/4
7		Nausea/vomiting	1/2
8		Nausea/vomiting	3/4
9		Neuropathy	1/2
10		Neuropathy	3/4
11	Progressive disease		–

*AE* Adverse event, *FN* Febrile neutropenia

### Step 3: content validation

We validated the first draft scenarios’ content in two processes: ‘first review’ and ‘cognitive interview.’ First, we checked each draft scenario for external and internal validity (first review). Specifically, three oncology physicians and four oncology pharmacists checked the information obtained during the ‘literature review’ and ‘exploratory interview’ process for consistency with the first draft scenario, to identify any additional content to consider, and amount of text, readability, adequacy, and accuracy of the item categories. The scenario was revised after consultation and agreement with other oncology experts on the need for modification after oncology experts’ feedback. We also asked one QoL expert to review the first-draft scenarios; the QoL expert considered the feasibility of modifications and additions according to the survey feasibility and readability, for the general population. Thus, we produced second-draft scenarios, and their SD and PD descriptions were divided into five categories: ‘summary,’ ‘physical symptoms,’ ‘daily life at home and outside,’ ‘mental’ and ‘AE.’ For ‘physical symptoms,’ appetite, fatigue, and body aches were selected, as they are considered the most frequent symptoms of distant MPC and have a significant impact on HRQoL.

We then conducted ‘cognitive interview’ to check the readability and comprehensibility of the second-draft scenarios among five individuals who were interviewed separately and who checked the meaning and degree of understanding of the scenarios. They answered questions including ‘What do you think the situation is?’ or ‘Can you imagine the situation in the scenario?’ We then interviewed them about aspects of the scenarios that deviated from what they imagined or that were not understandable. Finally, based on the ‘cognitive interview,’ we discussed the pros and cons of modifying the health status scenarios with three oncology physicians, four oncology pharmacists and a QoL expert, before developing the final survey scenario version (Supplementary Table 1).

### Step 4: utility research

Responders, the general Japanese population, excluded medical professionals (physicians, dentists, pharmacists, and nurses), were aged ≥20 years, and provided informed consent. Responders were recruited by a research contractor (Intage Healthcare Ltd. Tokyo. Japan) using snowball sampling method. Given the ongoing coronavirus disease 2019 (COVID-19) situation, we recruited participants only from the Kanto area, primarily Tokyo. Since gender and age could affect HRQoL, the sex ratio and age categories of respondents were similar.

Using face-to-face approach, the interviewers, who were trained in advance to minimize interviewer influence on the responders, explained the survey and provided instructions to respondents. First, respondents provided informed consent, background information (sex, age, employment status, education, and marital status), and three example questions (assessment of either wheelchair status, better than wheelchair status, or worse than dead status) that used cTTO, on a computer. Second, respondents answered the cTTO question in the eleven or six survey scenarios. The cTTO compared a certain number of years to live (as imagined by the respondents of their state of full heath [State A, which varies from 0 [to die now] to 10 years]) with 10 years to live in the presented survey scenario (State B), depending on their responses. Changes in the number of years of survival in State A were determined using a ping-pong approach. If respondents indicated that living in State B was more painful (worse than death now), they were moved to lead-time TTO (LT-TTO), which compares living a certain number of years in State A to 10 years in States A and B, respectively, that is, 20 years combined (Supplementary Fig. 1 shows the screens used in this study). Third, each survey scenario was presented again, and respondents assigned a score from 0 to 100, using the paper survey form of the VAS scale on ‘own health,’ ‘state of death,’ and ‘state of each scenario’. It is noteworthy that the ‘state of death’ was not necessarily zero. Finally, a three-question questionnaire was administered to ascertain the extent of understanding of the methods and scenarios, using a four-point rating (strongly agree, agree, disagree, and strongly disagree).

### Statistical analysis

This study aimed to clarify the utility value of MPC in Japanese general population by assessing MPC scenarios using cTTO. The summary statistics of population utility values were calculated using cTTO and VAS, rather than by setting hypothesis testing using some statistical methods. Furthermore, using individual-level utility scores from cTTO, we performed multiple regression analysis of fixed effect model to calculate health state valuations for each scenario. Because all explanatory variables were not orthogonal in this analysis, we only assessed the impact of independent adverse effect items on QOL for each health state and SD.

As we did not perform hypothesis testing, we did not calculate an exact sample size, but based on previous studies by Nafee et al., we considered that an interpretable summary statistic could be calculated from approximately 100 respondents’ data [41]. Similar to a previous study, the NSCLC QOL survey of the UK general population utility value variations per research scenario aggregated from 99 individuals’ data was low (SD range = 0.22–0.29). We were concerned about the impact of the number of scenarios in this study, on the results; therefore, we first surveyed 105 people with six pre-selected, randomly chosen scenarios. We then surveyed 105 individuals using all the 11 scenarios. The results for all surveyed people were combined for analysis.

We also excluded respondents who met any one of the following three exclusion criteria: respondents with incomplete answers, who were inconsistent in cTTO exercises 1 and 2, i.e., ‘the utility value calculated in exercise 2 was higher than the utility value calculated in exercise 1’; and those with utility scores = − 1, 0, or 1. Furthermore, to ascertain engagement with the responses, we tabulated the number of scenarios with a utility score of − 1, 0, or 1 and the response time per scenario and per question.

At sensitivity analysis, we calculated the utility value of each scenario by cTTO using the correction method used in a previous study by Nafee et al. [41] to change the negative QOL score to 0.02.

## Results

### Participant background

**Fig. 2:**
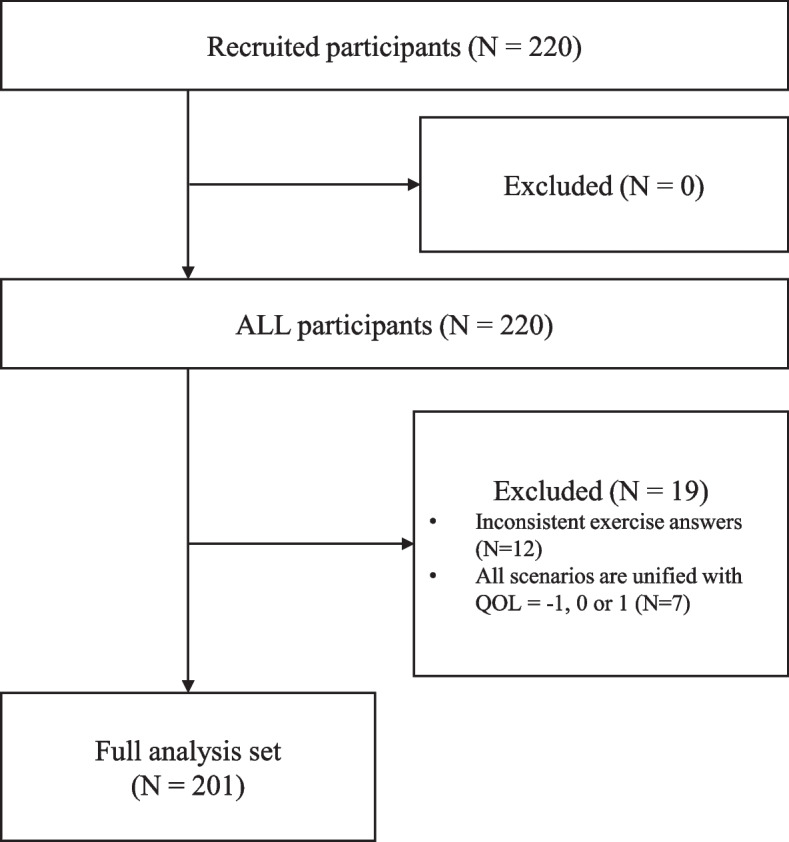
Participants flow diagram. QOL, quality of life

Of the 220 applicants who answered all questions (Fig. 2), 201 qualified for the analysis based on the eligibility criteria (excluding 19 [12 with inconsistent answers to cTTO exercises and 7 with scenarios’ utility scores equal to − 1, 0, or 1]). The results were reported by sex, age, employment status, education, and marital status in Table 2. The sex ratio and age distribution were homogeneous across the groups, and 59.7, 46.8, and 60.2% respondents were full-time employees, university graduates, and married, respectively.

**Table 2:** Participant characteristics

Characteristics	N	%
Gender		
Male	99	49.3%
Female	102	50.7%
Age (years)		
20–29	40	19.9%
30–39	37	18.4%
40–49	40	19.9%
50–59	41	20.4%
60–64	43	21.4%
Employment status		
Full-time employee	120	59.7%
Temporary worker	13	6.5%
Part-time worker	26	12.9%
Self-employed	8	4.0%
Unemployed	22	10.9%
Retired	1	0.5%
Student	11	5.5%
Other	0	0.0%
Education		
Junior high school	1	0.5%
Senior high school	51	25.4%
Vocational school / technical college	34	16.9%
Junior college	13	6.5%
University	94	46.8%
Master’s Degree	8	4.0%
Other	0	0.0%
Marital status		
Unmarried	65	32.3%
Married	121	60.2%
Other	15	7.5%

### Health statue utility values

The utility values for each survey scenario by cTTO and VAS, presented in Table 3, shows that the number of responses per scenario in the analysis population was more than 105 people. In the first half of the survey, 201 people responded to scenarios SD (reference), SD + neutropenia G1/2, SD + febrile neutropenia (FN) G3/4, SD + diarrhea G3/4, SD + vomiting G1/2, SD + neuropathy G3/4, and PD. Throughout all health status scenarios, the mean utility values in cTTO were lower than the median, whereas in VAS, they were generally comparable. Considering the mean utility values for each health status scenario, SD (cTTO, 0.634; VAS, 0.468) and SD + neutropenia G1/2 (cTTO,0.649; VAS,0.438) had the highest utility values for both cTTO and VAS. In contrast, the health status with the lowest mean utility value was PD (cTTO: -0.119, VAS:0.128) for cTTO and VAS. Health status with the lowest mean utility value among AEs, was SD + vomiting G3/4 (cTTO, 0.242; VAS, 0.193) for cTTO and SD + diarrhea G3/4 (cTTO, 0.306; VAS, 0.189). According to the distribution of utility values calculated from cTTO in SD and PD, < 5 and 22%of participants had a utility score of − 1 for SD and PD, respectively (Supplementary Fig. 2). Multiple regression analysis showed that among the AEs occurring in SD, FN G3/4, diarrhea G3/4, nausea/vomiting G1/2, nausea/vomiting G3/4, and neuropathy G3/4 significantly decreased utility values for SD (Supplementary Table 2). Moreover, sensitivity analysis results showed the largest change in utility values with PD compared to the base case analysis, with a utility value of 0.17, when negative utility values were replaced by 0.02 (Supplementary Table 3).

**Table 3:** Utility values for all health status of metastatic pancreas cancer from cTTO and VAS

Health status	AE grade	cTTO			VAS		
		N	AVG	STE	N	AVG	STE
SD (reference)	–	201	0.634	0.024	105	0.468	0.019
SD + neutropenia	G1/2	201	0.649	0.026	105	0.438	0.019
SD + neutropenia	G3/4	105	0.514	0.042	105	0.346	0.019
SD + FN	G3/4	201	0.323	0.035	105	0.201	0.017
SD + diarrhea	G1/2	105	0.500	0.042	105	0.296	0.018
SD + diarrhea	G3/4	201	0.306	0.039	105	0.189	0.017
SD + vomiting	G1/2	201	0.422	0.035	105	0.299	0.018
SD + vomiting	G3/4	105	0.242	0.057	105	0.193	0.016
SD + neuropathy	G1/2	105	0.541	0.039	105	0.331	0.019
SD + neuropathy	G3/4	201	0.370	0.037	105	0.251	0.018
PD	–	201	-0.119	0.040	105	0.112	0.020
Myself (VAS score)	–	–	–	–	–	0.891	0.010
Death (VAS score)	–	–	–	–	–	0.128	0.011

*SD* Stable disease, *PD* Progressive disease, *cTTO* Composite time trade-off, *VAS* Visual analog scale, *AE* Adverse event, *FN* Febrile neutropenia, *AVG* Average, *STE* Standard error, *G* Grade

### Participant engagement

Table 4 presents the tabulated results of the survey comprehension questionnaires for 220 individuals. Most assigned ‘strongly agree’ or ‘agree’ to the three-question questionnaire. Only one person reported that the poorest health PD on cTTO was better than the best health SD, with a median (interquartile range [IQR]) time taken for each question of 94 (74–125) seconds and a mean time to answer one cTTO question (1 task) in one health scenario value (SDI) of 5.28 (1.96) seconds. In addition, only three health status scenarios were reported with a utility score of 1 in the cTTO survey results.

**Table 4:** Evaluation of participant’s engagement with the study

Contents	N	%
**All participants**	**220**	**–**
Questioner (conducted after answering cTTO and VAS)		
Q1, ‘I found it easy to imagine the scenarios I was asked’		
Strongly agree	101	45.9%
Agree	110	50.0%
Disagree	8	3.6%
Strongly disagree	1	0.5%
Q2, ‘I found it easy to tell the difference between the lives I was asked to think about’		
Strongly agree	147	66.8%
Agree	71	32.3%
Disagree	2	0.9%
Strongly disagree	0	0.0%
Q3, ‘I found it difficult to decide on my answers to the questions’		
Strongly agree	57	25.9%
Agree	117	53.2%
Disagree	44	20.0%
Strongly disagree	2	0.9%
**Analysis population**	**201**	**–**
Participants evaluated utility value of PD > SD	1	0.5%
Time per cTTO task in seconds, median (IQR)	94 (74–125)	
Times of Trade-offs in cTTO, mean (SD)	5.28 (1.96)	
**cTTO tasks**	**1827**	**–**
Utility score = −1	90	4.9%
Utility score = 0	238	13.0%
Utility score = 1	3	0.2%

*cTTO* Composite time trade off, *VAS* Visual analog scale, *PD* Progressive disease, *SD* Stable disease, *IQR* Interquartile range

## Discussion

We developed utility research scenarios for MPC based on VBM and conducted utility research using cTTO and VAS in Japanese general population to clarify the social preferences of disutility toward MPC and its treatment-related AEs. As metastatic pancreatic cancer is a severe disease and it is difficult to conduct a direct QOL survey on patients, a survey of the general population using VBM was conducted in this study. In addition, as the severity of the disease is assumed to be a QOL value of worse than death, the survey was conducted mainly using cTTO, which can also measure WTD status. This approach is similar to the QOL survey for neuroendocrine tumors, which has been cited as an example of VBM implementation [26, 30]. Therefore, we consider the adoption of cTTO for metastatic pancreatic cancer appropriate. The utility value of SD in MPC calculated by cTTO was similar to that of SD in metastatic breast cancer and metastatic NSCLC utility research (SD with no toxicity:0.715 and 0.653). It was also lower than the EQ-5D-5L-derived utility value (0.697–0.74) for patients with advanced PC before and after chemotherapy in Japan [32, 33]. It was also smaller than the EQ-5D-5L-derived utility value (0.697–0.74) for patients with advanced PC before and after chemotherapy in Japan [33]. MPC has a worse prognosis than other cancers, and is a more severe disease state than advanced PC. Therefore, SD utility value based on cTTO in the present study is consistent with those of previous studies.

Among the scenarios, AE with the smallest disutility for SD was neutropenia G1/2. The mean QOL difference in neutropenia G1/2 to SD was 0.015 (SD and SD + neutropenia in cTTO = 0.634 and 0.649), well below minimal clinically important difference (MCID) proposed by various methods (MCID range, 0.03 to 0.054, especially in oncology, 0.07 to 0.12) [42]. The only difference between the two scenarios was the presence of ‘a mild decrease in immunity.’ This suggests that respondents consider changes that affect only laboratory values to have little impact on utility value. AEs with the highest degree of disutility were G3/4 diarrhea and vomiting, facilitate easy understanding of the harm and physical burden in the daily lives of the general population, and the frequency of their occurrence was clearly described in the scenarios. This may have led to higher disutility than for other AEs. Multiple regression analysis results support the finding that G3/4 AEs reduced utility beyond the MCID. However, even minor AEs, such as G1/2 nausea/vomiting, significantly lowered the utility. This suggests that even minor AEs may affect the HRQoL of patients with MPC.

The utility value based on cTTO in PD was negative, indicating a worse PD health status than death (WTD) in our study. The SD and PD states utility value distributions in the cTTO showed that although both states could take values between − 1 and 1, most respondents reported negative values in the PD state than in the SD state. This suggests that many respondents consider the PD state to be WTD, regardless of the degree of differences in values (optimistic or pessimistic) between individuals with regard to the disease, and suggests the influence of the framing effect, in which utility values are concentrated at − 1.0. In contrast, we described the disease states in PD of distant MPC based on the VBM by category, including vignette elements, and we created scenarios that helped respondents imagine the disease situations. The participant engagement results confirmed that the respondents were able to adequately imagine the content of the scenarios. Nevertheless, the representation of severe physical and psychological effects in the PD condition may have strongly influenced an increase in disutility. Additionally, in a study of EQ-5D hypothetical health status assessment by VAS, TTO, and SG in the general population in Japan, TTO tended to produce lower utility values than SG in assessing severe disease status, suggesting an influence on utility values between methods [43]. This also suggests that although the utility values in PD may have greater disutility due to the methodological limitations of cTTO, they are not necessarily uninterpretable and reflect the very severe disease state of PD in MPC.

The utility values from cTTO and VAS for each scenario were unequal. The utility values from cTTO ranged from the upper to lower limits of possible values for most health status scenarios, with a wide range of preferences per respondent, even for the same health status. That of VAS ranged far less between respondents than for cTTO, suggesting that visual ceiling and floor effects during the response. Confirming the order of utility values for each scenario in both methods, both cTTO and VAS had direct impacts on physical function, with AEs that were more severe and experience of symptoms having greater impacts on utility values. The ranking of QOL in each scenario was also similar, with no order reversal above one. Therefore, regardless of the investigation method, the results suggest a similar trend in the order of utility values calculated from each scenario in this study.

Utility values obtained from this survey reflect how respondents’ imagination of how MPC would affect their HRQoL. Although evidence exists from previous utility studies in patients with advanced PC, no study has examined MPC and the impact of each AE. Moreover, it has been difficult to conduct large-scale QOL studies in patients with MPC, due to lower survival rates and more severe disease status than those of other cancers. Therefore, we developed a disease scenario incorporating the elements of a vignette based on a scenario development process consisting of literature review, exploratory interviews, and cognitive interviews. In developing the scenarios, the respondents were unaware of distant MPC as the disease state, to avoid bias due to preconceptions and fears about utility [44].

## Limitation

This study has several limitations. First, the utility relevance for each disease state domain was uncertain. While several previous studies have evaluated the impact of symptoms, AEs, treatment and attributes, on an attribute-by-attribute basis by isolating the utility impact of specific attributes in the VBM, this study could not conduct an attribute-by-attribute analysis because the domains for each study scenario were not orthogonal [40, 45]. In contrast, we evaluated the impact of AE type and extent, to provide evidence on the chemotherapy safety for distant MPC, which has been unclear in previous studies. Second, the impact of severe disease states survey scenarios that could become WTD on utility research using cTTO is uncertain. In this study, we asked the general population to evaluate a scenario of MPC, a disease state with a very poor life expectancy and physical symptoms, using a trade-off for survival. We consider that few of the general population have experienced such a near-death clinical condition, and it would have been difficult for them to imagine the scenarios in the study as if they had the condition. Moreover, we did not specify the disease name, MPC, to avoid bias due to impressions of MPC, which could increase uncertainty in interpreting the scenarios. To minimize these effects, we created disease state scenarios through various processes based on the VBM to communicate serious disease states as objectively as possible. Based on cTTO’s practice questions and the result of the comprehension questionnaire at the end of the survey, we considered that a good understanding of the scenarios and how to answer the utility research questions by the respondents. Third, it is possible that the utility value for PD in the present study may have overestimated disutility compared to PD for cancer in previous studies. The reason for this is that we consider our PD scenario to reflect the worst state in the PD condition. In practice, metastatic pancreatic cancer patients with PD do not always continue to have the conditions shown in the PD scenarios. In contrast, the PD condition in MPC is objectively very serious, assuming a life expectancy of only a few months. For these reasons, it is difficult to conclude the utility value of PD in MPCs solely from the results of this study, although the utility value of PD in this study is considered partly interpretable. In particular, the extrapolation potential of PD utility values to validity assessment and cost-effectiveness assessment, etc., needs further investigation through surveys of healthcare professionals familiar with the condition and a small number of patients. Finally, as the respondent population in the study in question was only 20–64 years old in Tokyo due to Covid-19, concerns about a lack of exterritorial validity in Japan can be assumed. However, age and gender were considered to have been sampled equally in this study. Regarding other basic attributes, according to the 2020 Labour Force Survey of Japan, “full-time employees” accounted for 56.5% of the total working population [46]. In addition, according to the 2020 Census, 55.6% were “married”, and 34.6% of those aged 15 and over were “university” [47, 48]. These proportions are generally consistent with the basic demographics of the respondent patients in this study, which also ensures their external validity.

In this study, we clarified the utility value of MPC in Japan and the impact of the type and degree of AEs on the utility value by conducting utility research in the general population based on VBM. In particular, the study revealed that both G3/4 and G1/2 AEs may have a significant clinical impact on utility values. Since MPC has a shorter survival compared to other cancer types and chemotherapy regimen developments such as molecular-targeted agents with fewer AEs are still in the developmental stages, it is important to evaluate AEs, even minor ones, that may affect HRQoL. In the Japanese guidelines for cost-effectiveness assessment, it is listed as “If it is difficult to directly collect QOL scores from patients, it is acceptable for the general people to evaluate the presented health scenario by standard gamble (SG), time trade-off (TTO), and discrete choice experiment (DCE).” in section 8.3.3 of 2.0 edition [49]. Therefore, this study results using cTTO are not only useful for safety evaluation, including AEs in PC patients undergoing chemotherapy in actual clinical practice, but also provide evidence that can be partially used as parameters of effectiveness in cost-utility analysis of chemotherapy for PC. In future, additional studies including some healthcare professionals and patients using similar methods, should be conducted, to confirm the robustness and consistency of the assessment results in serious disease states such as PD, where cTTO may be limited in assessing these in the general population.

## Conclusion

We clarified the health state utility values in the general population for MPC in Japan. Among the chemotherapy AEs, the direct impact on physical symptoms and respondents’ experience may strongly influence utility values. In addition, the effect on utilities should be considered both for serious and minor AEs.

## Supplementary Information

**Additional file 1.**

## Data Availability

Our data are available only to the Department of Public Health and Epidemiology, Meiji Pharmaceutical University.

## Abbreviations

AE: Adverse events
COVID-19: Coronavirus disease 2019
CTCAE: Common Terminology Criteria for Adverse Events
cTTO: Composite time trade-off
EORTC-QLQ: European Organization for Research and Treatment of Cancer Quality of Life
EQ-5D-5L: EuroQol 5 dimensions 5-level
EuroQoL: European quality of life
FN: Febrile neutropenia
HRQoL: Health-related quality of life
HUI: Health utilities index
LT-TTO: Lead-time time trade-off
MCID: Minimal clinically important difference
MOS: Medical outcomes study
MPC: Metastatic pancreatic cancer
NCCN: National Comprehensive Cancer Network
NSCLC: Non-small cell lung cancer
PBMs: Preference-based measures
PC: Pancreatic cancer
PD: Progressive disease
QALYs: Quality-adjusted life years
QOL: Quality of life
RS: Rating scale
SD: Stable disease
SF-36: Short form-36
SF-6D: Short form-6 dimensions
TTO: Time trade-off
VAS: Visual analog scale
VBM: Vignette-based methods
WTD: Worse than death

## References

[1] Ferlay J, Soerjomataram I, Dikshit R, Eser S, Mathers C, Rebelo M (2015). Cancer incidence and mortality worldwide: sources, methods and major patterns in GLOBOCAN 2012. Int J Cancer.

[2] Noone AM, Howlader N, Krapcho M, Miller D, Brest A, Yu M, et al. SEER Cancer statistics review, 1975-2015, National Cancer Institute. Bethesda, https://seer.cancer.gov/csr/1975_2015/, Based on November 2017 SEER data submission, posted to the SEER web site, April 2018, Accessed 20 July 2020.

[3] Cancer Registry and Statistics, Cancer Information Service, National Cancer Center, Japan. Cohort Life Table. http://ganjoho.jp/reg_stat/statistics/qa_words/cohort01.html Accessed 25 July 2019. [in Japanese].

[4] Foundation for promotion of cancer research. Cancer statistics in Japan 2019 https://ganjoho.jp/data/reg_stat/statistics/brochure/2019/cancer_statistics_2019_fig_E.pdf Accessed 20 July 2020. [in Japanese].

[5] Center for Cancer Control and Information Services, National Cancer Center. Monitoring of Cancer Incidence in Japan—Survival 2006–2008 Report. 2016. Available online: https://ganjoho.jp/data/en/professional/statistics/files/cancer_survival(1993-2008)E.xls [in Japanese].

[6] Shinichi E, Hiroki T, Hiroaki O, Takuji O, Akimasa N, Takashi H, et al. A digest of the Pancreatic Cancer Registry Report 2007, Suizo, 2008, Volume 23, Issue 2, Pages 105-123, Released on J-STAGE May 29, 2008 Available online: 10.2958/suizo.23.105 [in Japanese].

[7] Okusaka T, Nakamura M, Yoshida M, Kitano M, Uesaka K, Ito Y (2020). Clinical practice guidelines for pancreas Cancer 2019 from the Japan pancreas society: a synopsis. Pancreas..

[8] Tempero MA, Malafa MP, Al-Hawary M, Behrman SW, Benson AB, Cardin DB (2021). Pancreas adenocarcinoma, version 2.2021, NCCN clinical practice guidelines in oncology. J Natl Compr Cancer Netw.

[9] Vainio A, Auvinen A (1996). Prevalence of symptoms among patients with advanced cancer: an international collaborative study. Symptom prevalence group. J Pain Symptom Manag.

[10] Breen WG, Jethwa KR, Yu NY, Spears GM, Harmsen WS, Miller RC (2021). Patient-reported quality of life before and after Chemoradiation for intact pancreas Cancer: a prospective registry study. Pract. Radiat Oncol.

[11] Burris HA, Moore MJ, Andersen J, Green MR, Rothenberg ML, Modiano MR (1997). Improvements in survival and clinical benefit with gemcitabine as first-line therapy for patients with advanced pancreas cancer: a randomized trial. J Clin Oncol.

[12] Conroy T, Desseigne F, Ychou M, Bouché O, Guimbaud R, Bécouarn Y (2011). Groupe Tumeurs digestives of Unicancer; PRODIGE intergroup. FOLFIRINOX versus gemcitabine for metastatic pancreas cancer. N Engl J Med.

[13] Von Hoff DD, Ervin T, Arena FP, Chiorean EG, Infante J, Moore M (2013). Increased survival in pancreas cancer with nab-paclitaxel plus gemcitabine. N Engl J Med.

[14] Ueno H, Ioka T, Ikeda M, Ohkawa S, Yanagimoto H, Boku N (2013). Randomized phase III study of gemcitabine plus S-1, S-1 alone, or gemcitabine alone in patients with locally advanced and metastatic pancreas cancer in Japan and Taiwan: GEST study. J Clin Oncol.

[15] Coveler AL, Mizrahi J, Eastman B, Apisarnthanarax SJ, Dalal S, McNearney T, Pant S (2021). Precision promise consortium. Pancreas Cancer-Associated Pain Management. Oncologist.

[16] Hendifar AE, Petzel MQB, Zimmers TA, Denlinger CS, Matrisian LM, Picozzi VJ, Rahib L (2019). Precision promise consortium. Pancreas Cancer-Associated Weight Loss. Oncologist.

[17] Goldberg RJ, Cullen LO (1986). Depression in geriatric cancer patients: guide to assessment and treatment. Hosp J.

[18] Carelle N, Piotto E, Bellanger A, Germanaud J, Thuillier A, Khayat D (2002). Changing patient perceptions of the side effects of cancer chemotherapy. Cancer..

[19] Bauer MR, Bright EE, MacDonald JJ, Cleary EH, Hines OJ, Stanton AL (2018). Quality of life in patients with pancreas Cancer and their caregivers: a systematic review. Pancreas..

[20] Neumann PJ, Ganiats TG, Russell LB, Sanders GD, Siegel JE (2017). Cost-effectiveness in health and medicine.

[21] Ware JE, Sherbourne CD (1992). The MOS 36-item short-form health survey (SF-36). I. Conceptual framework and item selection. Med Care.

[22] Fayers P, Aaronson NK, Bjordal K, Groenvold M, Curran D, Bottomley A (2001). EORTC QLQ-C30 Scoring Manual. (3rd ed.) European Organisation for Research and Treatment of Cancer.

[23] Herdman M, Gudex C, Lloyd A, Janssen M, Kind P, Parkin D (2011). Development and preliminary testing of the new five-level version of EQ-5D (EQ-5D-5L). Qual Life Res.

[24] Brazier J, Jones N, Kind P (1993). Testing the validity of the Euroqol and comparing it with the SF-36 health survey questionnaire. Qual Life Res.

[25] Horsman J, Furlong W, Feeny D, Torrance G (2003). The health utilities index (HUI): concepts, measurement properties and applications. Health Qual Life Outcomes.

[26] Matza LS, Stewart KD, Lloyd AJ, Rowen D, Brazier JE (2021). Vignette-based utilities: usefulness, limitations, and methodological recommendations. Value Health.

[27] Von Neumann J, Morgenstern O (1944). Theory of games and economic behavior.

[28] Torrance GW (1970). A generalized cost-effectiveness model for the evaluation of health programs.

[29] Lloyd A, Piglowska N, Ciulla T, Pitluck S, Johnson S, Buessing M, O'Connell T (2019). Estimation of impact of RPE65-mediated inherited retinal disease on quality of life and the potential benefits of gene therapy. Br J Ophthalmol.

[30] Swinburn P, Wang J, Chandiwana D, Mansoor W, Lloyd A (2012). Elicitation of health state utilities in neuroendocrine tumours. J Med Econ.

[31] Matza LS, Stewart KD, Gandra SR, Delio PR, Fenster BE, Davies EW (2015). Acute and chronic impact of cardiovascular events on health state utilities. BMC Health Serv Res.

[32] Hagiwara Y, Ohashi Y, Uesaka K (2018). Health-related quality of life of adjuvant chemotherapy with S-1 versus gemcitabine for resected pancreas cancer: results from a randomised phase III trial (JASPAC 01). Eur J Cancer.

[33] Fujii H, Koda M, Sadaka S (2021). Anorexia, pain and peripheral neuropathy are associated with a decrease in quality of life in patients with advanced pancreas cancer receiving outpatient chemotherapy - a retrospective observational study. J Pharm Health Care Sci.

[34] Patrick DL, Burke LB, Gwaltney CJ, Leidy NK, Martin ML, Molsen E, Ring L (2011). Content validity--establishing and reporting the evidence in newly developed patient-reported outcomes (PRO) instruments for medical product evaluation: ISPOR PRO good research practices task force report: part 1--eliciting concepts for a new PRO instrument. Value Health.

[35] Patrick DL, Burke LB, Gwaltney CJ, Leidy NK, Martin ML, Molsen E, Ring L (2011). Content validity--establishing and reporting the evidence in newly developed patient-reported outcomes (PRO) instruments for medical product evaluation: ISPOR PRO Good Research Practices Task Force report: part 2--assessing respondent understanding. Value Health.

[36] Sharma C, Eltawil KM, Renfrew PD, Walsh MJ, Molinari M (2011). Advances in diagnosis, treatment and palliation of pancreas carcinoma: 1990-2010. World J Gastroenterol.

[37] Common terminology criteria for adverse events (CTCAE) version 4.03. 2010. Japan clinical oncology group. http://www.jcog.jp/doctor/tool/CTCAEv4J_20170912_v20_1.pdf [in Japanese] 15818867

[38] Common terminology criteria for adverse events (CTCAE) version 5.0. 2017. Japan clinical oncology group. http://www.jcog.jp/doctor/tool/CTCAEv5J_20210901_v24_1.pdf [in Japanese]

[39] Aaronson NK, Ahmedzai S, Bergman B, Bullinger M, Cull A, Duez NJ (1993). The European Organization for Research and Treatment of Cancer QLQ-C30: a quality-of-life instrument for use in international clinical trials in oncology. J Natl Cancer Inst.

[40] Lloyd A, Nafees B, Narewska J, Dewilde S, Watkins J (2006). Health state utilities for metastatic breast cancer. Br J Cancer.

[41] Nafees B, Stafford M, Gavriel S, Bhalla S, Watkins J (2008). Health state utilities for non small cell lung cancer. Health Qual Life Outcomes.

[42] Coretti S, Ruggeri M, McNamee P (2014). The minimum clinically important difference for EQ-5D index: a critical review. Expert Rev Pharmacoecon Outcomes Res.

[43] Sakai I, Fukuda T, Tamura M, Mori K, Tsuchiya Y, Ikeda S. The Relationship among Values of Different Health Status Measures. Iryo To Shakai, 1998–1999, 8, No.1, p79–93. Available online: 10.4091/iken1991.8.1_79 [in Japanese]

[44] Rowen D, Brazier J, Tsuchiya A, Young T, Ibbotson R (2012). It's all in the name, or is it? The impact of labeling on health state values. Med Decis Mak.

[45] Fordham BA, Kerr C, de Freitas HM, Lloyd AJ, Johnston K, Pelletier CL (2015). Health state utility valuation in radioactive iodine-refractory differentiated thyroid cancer. Patient Prefer Adherence.

[46] Labour Force Survey, Statistics Bureau of the Ministry of Internal Affairs and Communications Summary of past results. https://www.stat.go.jp/data/roudou/rireki/nen/ft/pdf/2020, Page 8, 13. [in Japanese]

[47] 2020 Census, population and other basic tabulation results, summary of results. https://www.stat.go.jp/data/kokusei/2020/kekka/pdf/outline_01.pdf, Page 28. [in Japanese]

[48] 2020 Census, Basic tabulation of employment status, etc. (main contents: labour force status, industry and occupation of workers, education, etc.). https://www.e-stat.go.jp/stat-search/file-download?statInfId=000032201216&fileKind=0 [in Japanese]

[49] Center for Outcomes Research and Economic Evaluation for Health, Guideline for cost-effectiveness evaluation in Japan (second edition) [English], https://c2h.niph.go.jp/tools/guideline/guideline_en.pdf.

