# Correction: Health state utility values for metastatic pancreatic cancer using a composite time trade-off based on the vignette-based approach in Japan

**DOI:** 10.1186/s13561-023-00418-x

**Published:** 2023-01-11

**Authors:** Yuki Takumoto, Yuriko Sasahara, Hiroto Narimatsu, Tatsunori Murata, Manabu Akazawa

**Affiliations:** 1 Department of Public Health and Epidemiology, Meiji Pharmaceutical University, 2-522-1 Noshio, Kiyose, Tokyo Japan; 2 Center for Outcomes Research and Economic Evaluation for Health, National Institute of Public Health, Saitama, Japan; 3 Department of Medical Oncology, Yamagata Prefectural Central Hospital, Yamagata, Japan; 4 Department of Genetic Medicine, Kanagawa Cancer Center, Yokohama, Kanagawa Japan; 5 Cancer Prevention and Cancer Control Division, Kanagawa Cancer Center Research Institute, Yokohama, Kanagawa Japan; 6 Graduate School of Health Innovation, Kanagawa University of Human Services, Yokohama, Kanagawa Japan; 7 CRECON Medical Assessment Inc, Tokyo, Japan

**Correction: Health Econ Rev 12, 63 (2022)**

10.1186/s13561-022-00413-8

Following publication of the original article [1], the authors identified errors in the names of all authors. The given name and family name were erroneously transposed, and the given name of “Hiroto Narimatsu” was incorrectly listed as “Hirohito”.

The author group has been updated above and the original article [1] has been corrected.

## References

[1] Takumoto Y, Sasahara Y, Narimatsu H (2022). Health state utility values for metastatic pancreatic cancer using a composite time trade-off based on the vignette-based approach in Japan. Health Econ Rev.

