# Supplementary material for: Health state utility values for metastatic pancreatic cancer using a composite time trade-off based on the vignette-based approach in Japan
Source: Health Econ Rev. 2022 Dec 24;12:63. doi: 10.1186/s13561-022-00413-8 (PMC9789314; doi:10.1186/s13561-022-00413-8)
Supplement: Supplementary file 1 — Additional file 1. [file 13561_2022_413_MOESM1_ESM.pdf]

**Supplementary Material**

Authors:

Y Takumoto, Y Sasahara, H Narimatsu, T Murata and M Akazawa

Title: Health state utility values for metastatic pancreatic cancer using a time trade-off based on a vignette-based approach in Japan

eTable 1: Disease state scenarios of patients with pancreatic cancer (English translation).

eTable 2: Results of multiple regression analysis with fixed effects

eTable 3: Comparison of the results of the sensitivity analysis and base case analysis in cTTO

eFigure 1: Example of a display screen in a composite time trade-off (English translation)

eFigure 2: Distribution of utility values based on cTTO for SD and PD scenarios

eTable 1: Disease state scenarios of patients with pancreatic cancer (English translation).

| No | Health status | Classification | | Contents |
|---|---|---|---|---|
| 1 | SD | Summary | | You have a life-threatening disease, but his condition has been stabilized by treatment. |
| | | Physical symptoms | Appetite | You do not have enough appetite to lose weight. |
| | | | Fatigue | You are more tired than when you were healthy. |
| | | | Body aches | Sometimes you have strong pain. You take medication to relieve it. |
| | | Daily life at home and outside | | You can live by yourself at home, but you can only go out for a couple of hours. |
| | | Mental | | You are very anxious about the possibility of dying from the disease. |
| | | Adverse effects | | None. |
| 2 | SD + Neutropenia Grade1/2 | Summary | | You have a life-threatening disease, but his condition has been stabilized by treatment. |
| | | Physical symptoms | Appetite | You do not have enough appetite to lose weight. |
| | | | Fatigue | You are more tired than when you were healthy. |
| | | | Body aches | Sometimes you have strong pain. You take medication to relieve it. |
| | | Daily life at home and outside | | You can live by yourself at home, but you can only go out for a couple of hours. |
| | | Mental | | You are very anxious about the possibility of dying from the disease. |
| | | Adverse effects | | As an adverse event of treatment, you have a mild decrease in immunity, but you do not have any physical symptoms such as lethargy or fever. |
| 3 | SD + Neutropenia Grade3/4 | Summary | | You have a life-threatening disease, but his condition has been stabilized by treatment. |
| | | Physical symptoms | Appetite | You do not have enough appetite to lose weight. |
| | | | Fatigue | You are more tired than when you were healthy. |
| | | | Body aches | Sometimes you have strong pain. You take medication to relieve it. |

| | | | | |
|---|---|---|---|---|
| | | Daily life at home and outside | | You can live by yourself at home, but you can only go out for a couple of hours. |
| | | Mental | | You are very anxious about the possibility of dying from the disease. |
| | | Adverse effects | | As an adverse event of treatment, your immune system is compromised.<br>You do not have any physical symptoms such as lethargy or fever, but you have to refrain from going out due to the increased risk of infection. |
| 4 | SD +<br>FN<br>Grade3/4 | Summary | | You have a life-threatening disease, but his condition has been stabilized by treatment. |
| | | Physical symptoms | Appetite | You do not have enough appetite to lose weight. |
| | | | Fatigue | You are more tired than when you were healthy. |
| | | | Body aches | Sometimes you have strong pain. You take medication to relieve it. |
| | | Daily life at home and outside | | You can live by yourself at home, but you can only go out for a couple of hours. |
| | | Mental | | You are very anxious about the possibility of dying from the disease. |
| | | Adverse effects | | As the adverse event of treatment, you have lethargy and a fever over 38 degrees, requiring urgent hospitalization (intravenous fluids, etc.). |
| 5 | SD +<br>Diarrhea<br>Grade1/2 | Summary | | You have a life-threatening disease, but his condition has been stabilized by treatment. |
| | | Physical symptoms | Appetite | You do not have enough appetite to lose weight. |
| | | | Fatigue | You are more tired than when you were healthy. |
| | | | Body aches | Sometimes you have strong pain. You take medication to relieve it. |
| | | Daily life at home and outside | | You can live by yourself at home, but you can only go out for a couple of hours. |
| | | Mental | | You are very anxious about the possibility of dying from the disease. |

| | | | | |
|---|---|---|---|---|
| | | Adverse effects | | As an adverse event of treatment, you have 4 to 6 more bowel movements and diarrhea per day than usual. |
| 6 | SD + Diarrhea Grade3/4 | Summary | | You have a life-threatening disease, but his condition has been stabilized by treatment. |
| | | Physical symptoms | Appetite | You do not have enough appetite to lose weight. |
| | | | Fatigue | You are more tired than when you were healthy. |
| | | | Body aches | Sometimes you have strong pain. You take medication to relieve it. |
| | | Daily life at home and outside | | You can live by yourself at home, but you can only go out for a couple of hours. |
| | | Mental | | You are very anxious about the possibility of dying from the disease. |
| | | Adverse effects | | As an adverse event of treatment, you have diarrhea more than seven times a day, which requires hospitalization (intravenous fluids, etc.). |
| 7 | SD + Nausea/ Vomiting Grade1/2 | Summary | | You have a life-threatening disease, but his condition has been stabilized by treatment. |
| | | Physical symptoms | Appetite | You do not have enough appetite to lose weight. |
| | | | Fatigue | You are more tired than when you were healthy. |
| | | | Body aches | Sometimes you have strong pain. You take medication to relieve it. |
| | | Daily life at home and outside | | You can live by yourself at home, but you can only go out for a couple of hours. |
| | | Mental | | You are very anxious about the possibility of dying from the disease. |
| | | Adverse effects | | As an adverse event of treatment, you have nausea and vomiting 2~3 times a day. |
| 8 | SD + Nausea/ Vomiting Grade3/4 | Summary | | You have a life-threatening disease, but his condition has been stabilized by treatment. |
| | | Physical symptoms | Appetite | You do not have enough appetite to lose weight. |
| | | | Fatigue | You are more tired than when you were healthy. |

| | | | | |
|---|---|---|---|---|
| | | | Body aches | Sometimes you have strong pain. You take medication to relieve it. |
| | | Daily life at home and outside | | You can live by yourself at home, but you can only go out for a couple of hours. |
| | | Mental | | You are very anxious about the possibility of dying from the disease. |
| | | Adverse effects | | As an adverse event of treatment, you have nausea and vomiting 4~6 times a day. |
| 9 | SD + Neuropathy Grade1/2 | Summary | | You have a life-threatening disease, but his condition has been stabilized by treatment. |
| | | Physical symptoms | Appetite | You do not have enough appetite to lose weight. |
| | | | Fatigue | You are more tired than when you were healthy. |
| | | | Body aches | Sometimes you have strong pain. You take medication to relieve it. |
| | | Daily life at home and outside | | You can live by yourself at home, but you can only go out for a couple of hours. |
| | | Mental | | You are very anxious about the possibility of dying from the disease. |
| | | Adverse effects | | As an adverse event of treatment, you have persistent tingling pain and numbness in your hands and feet, which is uncomfortable. |
| 10 | SD + Neuropathy Grade3/4 | Summary | | You have a life-threatening disease, but his condition has been stabilized by treatment. |
| | | Physical symptoms | Appetite | You do not have enough appetite to lose weight. |
| | | | Fatigue | You are more tired than when you were healthy. |
| | | | Body aches | Sometimes you have strong pain. You take medication to relieve it. |
| | | Daily life at home and outside | | You can live by yourself at home, but you can only go out for a couple of hours. |
| | | Mental | | You are very anxious about the possibility of dying from the disease. |

| | | | | |
|---|---|---|---|---|
| | | Adverse effects | | As an adverse event of treatment, you have severe numbness in your hands and feet, making it difficult to button or grip small objects, and when it is terrible, it is difficult to walk. |
| 11 | PD | Summary | | You have a life-threatening disease. We have tried everything to treat you, but your condition is worsening. |
| | | Physical symptoms | Appetite | You have lost your appetite to the point of changing your body shape. |
| | | | Fatigue | You feel exhausted and lazy all the time. |
| | | | Body aches | You have severe pain in your stomach and back that does not go away with painkillers. |
| | | Daily life at home and outside | | You need nursing care for all your daily activities. |
| | | Mental | | You have a constant fear of death and intense anxiety about leaving your family behind. |
| | | Adverse effects | | None. |

SD, stable disease; PD, progressive disease; FN, febrile neutropenia

eTable 2: Results of multiple regression analysis with fixed effects

| Parameter | Estimate | STE | t-value | P-value |
|---|---|---|---|---|
| Intercept (SD) | 0.633 | 0.039 | 16.41 | <.0001 |
| Neutropenia G1/2 | 0.015 | 0.048 | 0.31 | 0.7531 |
| Neutropenia G3/4 | -0.119 | 0.058 | -2.04 | 0.041 |
| FN G3/4 | -0.310 | 0.048 | -6.45 | <.0001 |
| Diarrhea G1/2 | -0.133 | 0.058 | -2.28 | 0.0228 |
| Diarrhea G3/4 | -0.328 | 0.048 | -6.79 | <.0001 |
| Nausea / Vomitting G1/2 | -0.211 | 0.048 | -4.39 | <.0001 |
| Nausea / Vomitting G3/4 | -0.392 | 0.058 | -6.7 | <.0001 |
| Neuropathy G1/2 | -0.092 | 0.058 | -1.58 | 0.1148 |
| Neuropathy G3/4 | -0.264 | 0.048 | -5.48 | <.0001 |

STE: standard error. This analysis included 201 participants.

eTable 3: Comparison of the results of the sensitivity analysis and base case analysis in cTTO

| Health status | AE Grade | N | Base case analysis | | Sensitive analysis | | Difference |
|---|---|---|---|---|---|---|---|
| | | | AVG | STE | AVG | STE | |
| SD (Reference) | - | 201 | 0.634 | 0.024 | 0.642 | 0.023 | -0.008 |
| SD + Neutropenia | G1/2 | 201 | 0.649 | 0.026 | 0.668 | 0.022 | -0.019 |
| SD + Neutropenia | G3/4 | 105 | 0.514 | 0.042 | 0.55 | 0.033 | -0.036 |
| SD + FN | G3/4 | 201 | 0.323 | 0.035 | 0.406 | 0.023 | -0.083 |
| SD + Diarrhea | G1/2 | 105 | 0.5 | 0.042 | 0.541 | 0.032 | -0.041 |
| SD + Diarrhea | G3/4 | 201 | 0.306 | 0.039 | 0.433 | 0.023 | -0.127 |
| SD + Nausea/Vomiting | G1/2 | 201 | 0.422 | 0.035 | 0.5 | 0.023 | -0.078 |
| SD + Nausea/Vomiting | G3/4 | 105 | 0.242 | 0.057 | 0.396 | 0.032 | -0.154 |
| SD +Neuropathy | G1/2 | 105 | 0.541 | 0.039 | 0.571 | 0.03 | -0.03 |
| SD +Neuropathy | G3/4 | 201 | 0.37 | 0.037 | 0.465 | 0.024 | -0.095 |
| PD | - | 201 | -0.119 | 0.04 | 0.171 | 0.018 | -0.29 |

AVG: average; STE: standard error; cTTO, composite time trade-off; SD, stable disease; PD, progressive disease; FN, febrile neutropenia.

eFigure 1: Example of a display screen in a composite time trade-off (English translation)

| QX | You will be asked to think about the "Full health" and the "Impaired health" shown below.<br>After reading all the statements on the screen, imagine how you would feel if you were in such a health condition. |
|---|---|

| | | Full health | Impaired health |
|---|---|---|---|
| Summary | | Physically and mentally, the healthiest state. | You have a life-threatening disease, but his condition has been stabilized by treatment. |
| Physical symptoms | Appetite | You have the same appetite as usual. | You do not have enough appetite to lose weight. |
| | Fatigue | You can eating as usual. | You are more tired than when you were healthy. |
| | Body aches | You have not pain. | Sometimes you have strong pain. You take medication to relieve it. |
| Daily life at home and outside | | You can live freely on your own. | You can live by yourself at home, but you can only go out for a couple of hours. |
| Mental | | You have no worries or concerns about your health. | You are very anxious about the possibility of dying from the disease. |
| Adverse effects | | None. | As an adverse event of treatment, you have nausea and vomiting 2~3 times a day. |

| QX_yy | We created two cases by combining two health conditions and years of survival.<br>When do you imagine the two cases in your own life,<br>which one you think is more preferable for you, Case 1 or Case 2, as shown below? |
|---|---|

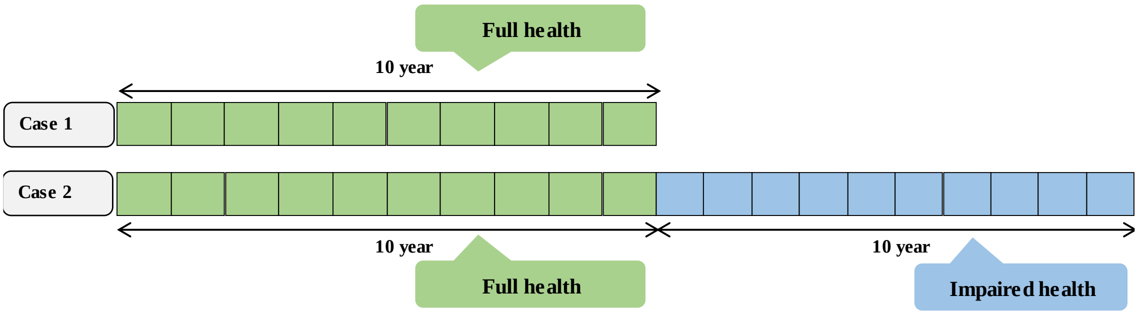


- Case 1: Live only 10 years in "perfect health".
- Case 1 and Case 2 are the same for you.
- Case 2: Live for 10 years in "perfect health" and then live for 10 years in the "specific health condition" described above.

eFigure 2: Distribution of utility values based on cTTO for SD and PD scenarios

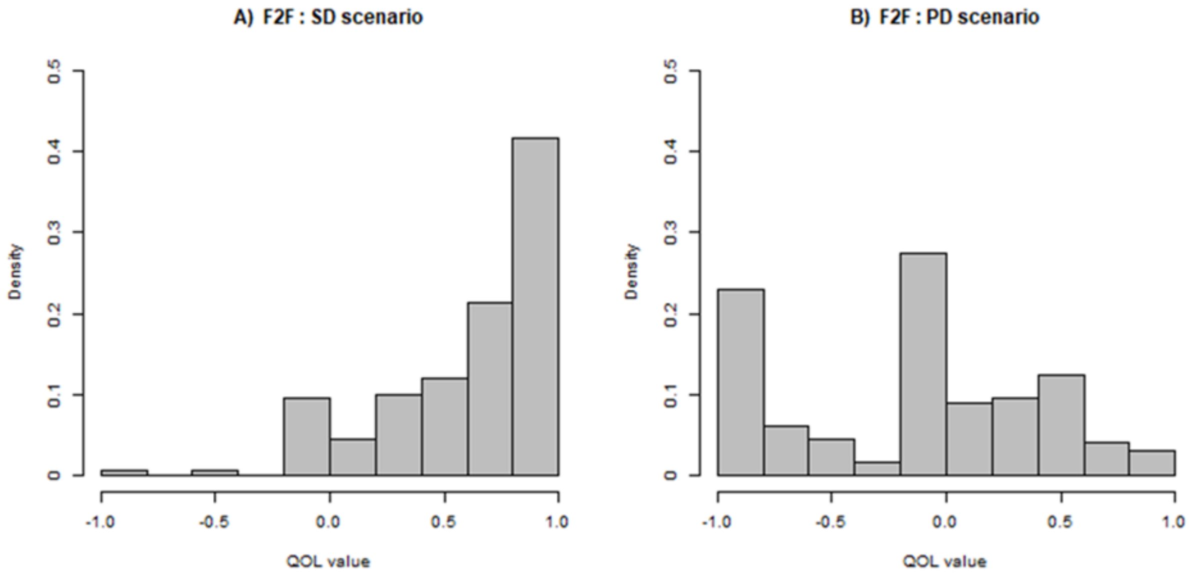


cTTO, composite time trade-off; F2F: face-to-face; SD: stable disease; PD: progressive disease; QOL, quality of life
